# Impact of surface wettability on S-layer recrystallization: a real-time characterization by QCM-D

**DOI:** 10.3762/bjnano.8.10

**Published:** 2017-01-11

**Authors:** Jagoba Iturri, Ana C Vianna, Alberto Moreno-Cencerrado, Dietmar Pum, Uwe B Sleytr, José Luis Toca-Herrera

**Affiliations:** 1 Institute for Biophysics, Dept. of Nanobiotechnology, BOKU University for Natural Resources and Life Sciences, Muthgasse 11 (Simon Zeisel Haus), A-1190 Vienna, Austria; 2 University of Sao Paulo (USP), Faculty of Philosophy, Science and Letters of Ribeirao Preto (FFCLRP), Department of Chemistry, Ribeirao Preto, SP, Brazil

**Keywords:** bacterial S-layers, Quartz crystal microbalance with dissipation monitoring (QCM-D), recrystallization kinetics, surface wettability

## Abstract

Quartz crystal microbalance with dissipation monitoring (QCM-D) has been employed to study the assembly and recrystallization kinetics of isolated SbpA bacterial surface proteins onto silicon dioxide substrates of different surface wettability. Surface modification by UV/ozone oxidation or by vapor deposition of 1*H*,1*H*,2*H*,2*H*-perfluorododecyltrichlorosilane yielded hydrophilic or hydrophobic samples, respectively. Time evolution of frequency and dissipation factors, either individually or combined as the so-called *Df* plots, showed a much faster formation of crystalline coatings for hydrophobic samples, characterized by a phase-transition peak at around the 70% of the total mass adsorbed. This behavior has been proven to mimic, both in terms of kinetics and film assembly steps, the recrystallization taking place on an underlying secondary cell-wall polymer (SCWP) as found in bacteria. Complementary atomic force microscopy (AFM) experiments corroborate these findings and reveal the impact on the final structure achieved.

## Introduction

Crystalline bacterial protein layers (S-layers) are arrays of (glyco)proteins (*M*_w_ of 40 to 200 kDa) forming the outermost envelope of prokaryotes, and represent the simplest biological membrane [1–2]. Depending on the number of identical protein subunits forming each morphological unit, S-layers show oblique (p1, p2), square (p4), or hexagonal (p3, p6) lattice symmetries, with unit cell dimensions ranging from 3 to 30 nm [3]. More specifically, the S-layer protein employed in this work, the SbpA from *Lysinibacillus sphaericus CCM2177*, presents a square (p4) lattice symmetry with a reported spacing of around 13 nm and base angle of 90° in its natural environment [4].

**Figure 1:**
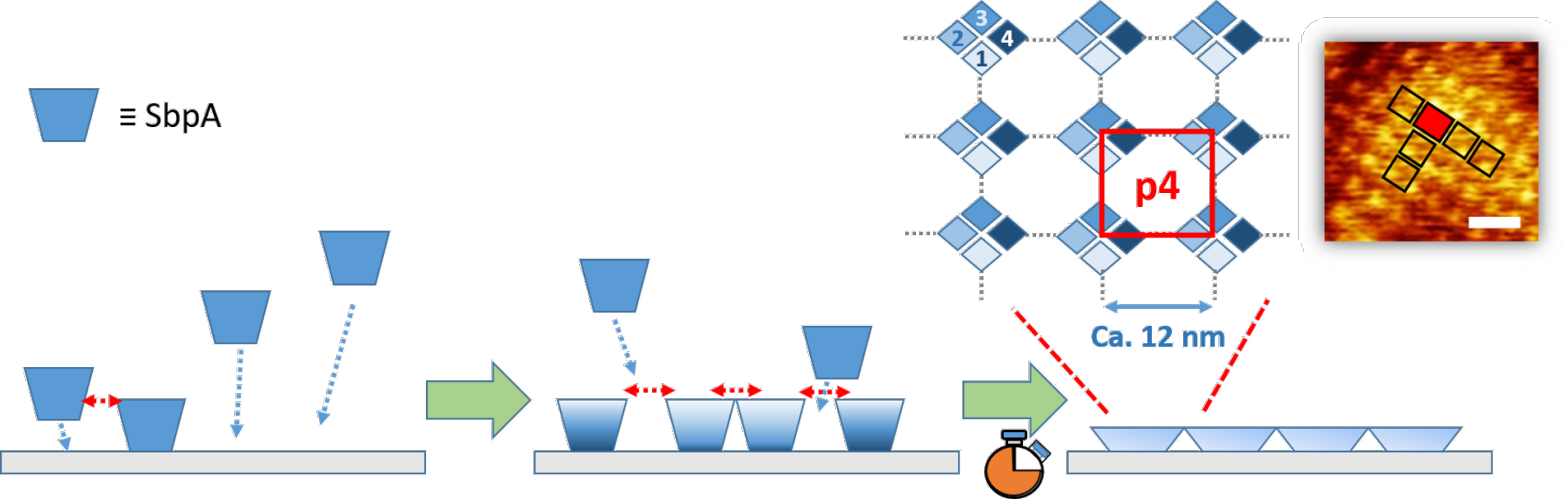
General scheme of SbpA protein S-layer crystal formation on a planar substrate: (left) the initial approach and binding of protein units to the surface co-exists with inter-monomeric interactions that lead to the formation of first nucleation points. Incorporation of additional building blocks takes place in parallel to the first crystal formation (center), which regulates the SbpA-film interacting capability throughout the process. After a certain incubation time, completion of the protein assembly leads to a full coverage of the surface (right). This film appears as a crystalline coating with regular square-like (p4) lattice, with four SbpA sub-units involved, as shown in the AFM height image. The scale bar corresponds to 25 nm.

From the point of view of bioengineering, it is highly interesting that isolated S-layer protein subunits have the ability to self-assemble, through the so-called recrystallization process, on a broad number of supports (see the general scheme in Figure 1). In former studies, the capability of SbpA to recrystallize from bulk has been tested on common bare substrates (silicon wafers, mica, gold) [5–9], with coatings of different nature (lipid bilayers, SAMs, polyelectrolyte multilayers) [10–14] and even on structures with an important contribution in the *Z*-axis, as recently reported for charged polyelectrolyte brushes [15]. However, some fundamental questions are yet to be solved about the S-layer recrystallization process under different physico-chemical conditions of the substrate (i.e., wettability or the presence of specific chemical groups and receptors), such as the different adsorption kinetics, crystal domain sizes and lattice symmetry parameters obtained. The importance of such factors for the morphology and biological function of isolated proteins has been already shown in literature [16].

In this regard, the quartz crystal microbalance with dissipation monitoring (QCM-D) has proven to be a powerful technique to follow in situ the binding of S-layers on different supports [17–18]. The QCM-D responses, i.e., the resonance frequency *f* and the energy dissipation *D* of the shear oscillatory motion of a piezoelectric quartz crystal sensor, change upon adsorption or desorption of material on the surface of that sensor. The measured parameters are highly sensitive to the mass (of the order of a few nanograms per square centimeter) and the mechanical properties of the surface-bound layer and can be monitored simultaneously and in real time. Conceptually, differences in frequency reflect interaction of the bacterial protein with the substrate, while dissipation changes are related to the structural rearrangement and variation of the viscous contribution of the film. The combination of both factors in the so-called *Df* plots (Δ*D* vs Δ*f*) enhances the information extracted, in comparison to the individual measurements, and has become a very useful tool to characterize the formation of biofilms in the recent years [19–23].

In this manuscript, our study is focused on a physico-chemical analysis about how changes in the wettability (hydrophilic vs hydrophobic) of SiO_2_ surfaces influence the recrystallization pathways of SbpA proteins. By means of either oxidative treatments (UV/ozone) or vapor deposition of a fluorinated silane the wetting properties of the substrate could be easily tailored to be hydrophilic or hydrophobic. Then, exposure of the samples to SbpA and their subsequent evolution in time was followed in situ by QCM-D with the support of atomic force microscopy for topographical analysis of the resulting crystal-like films. The results are compared with the biomimetic case found in bacteria represented by the interaction of SbpA with a secondary cell-wall polymer (SCWP), which specifically recognizes the N-terminal region of the S-layer protein [24–25].

## Results and Discussion

### S-layer formation on secondary cell-wall polymer (SCWP) films

**Figure 2:**
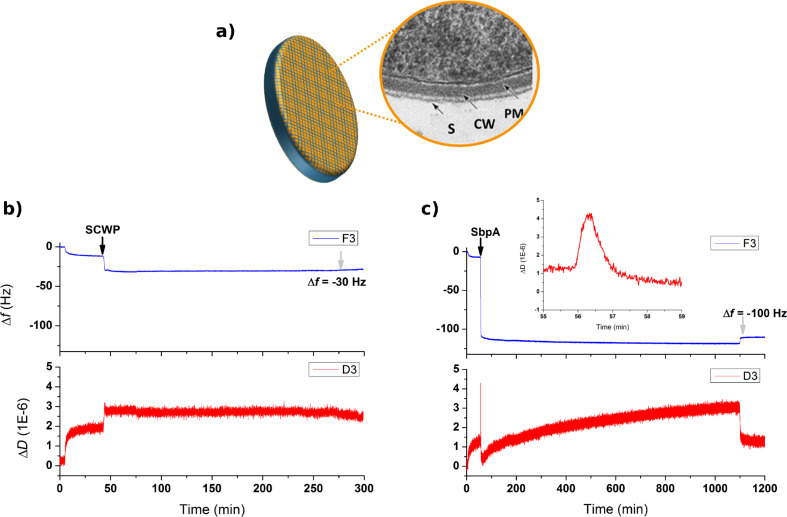
(a) Schematic view of a Gram positive bacterium with a magnified view of the surface structure shown as an electron micrograph of a thin section (adapted from [2]). Abbreviations indicate: Gram-positive cell wall (CW); plasma membrane (PM) and S-layer (S). Figures (b) and (c) show the real time variation of ∆*f* (in Hz) and ∆*D* (in units of 1 × 10^−6^) for the 3rd overtone as recorded for the injection of thiolated-SCWP on bare gold and SbpA protein on the preformed SCWP film, respectively. Black arrows indicate the exact injection time while grey arrows refer to the buffer rinse at the end of the process. The inset in (c) highlights the characteristic peak observed at the initial moments of the S-layer formation.

Both the binding of thiolated-SCWP to gold surfaces and its subsequent use as underlying material for the recrystallization of SbpA bacterial protein could be monitored in real time by means of QCM-D (Figure 2). As the first step, the in vitro formation of a SCWP film mimicking the underlying material found by bacterial S-layer proteins in nature (Figure 2a) induced a variation in frequency of 30 Hz, corresponding to a bound mass of about 0.53 µg/cm^2^ (Figure 2b). The low values measured for Δ*D* (below 1 × 10^−6^) hint at a very thin film that can approximately be calculated to be about 5–6 nm, and at a low water content of the film. This result agrees with what is expected from the internal arrangement of the polymer [25]. However, this slight modification is sufficient to provide a new character to the sensor.

Subsequently, the SCWP was exposed to a 0.1 mg/mL solution of SbpA in crystallization buffer (CB) and left to evolve overnight. As shown in Figure 2c this protein injection caused a sudden decrease in ∆*f* down to values of around −100 Hz. After that a plateau that remained throughout the incubation time (ca. 17 h) was reached quickly. The steadiness of the values derives from null mass adsorption over this period or, in other words, the full completion of the coating, which prevents further binding of SbpA units from the bulk. Final rinse with CB is employed to quench the incubation, causing no remarkable changes in the S-layer structure besides the flushing of unbound protein residues. The related variation in dissipation provides complementary information about the film formation process, since the drop in frequency is accompanied simultaneously by an increase in Δ*D* which, after a maximum, drops rapidly to much lower values. This peak appearance can be explained by a transitional step from mere protein adsorption (inducing an increase in Δ*D*) to a sudden stiffening of the film as SbpA keeps attaching. This behavior can be explained by the simultaneously ongoing recrystallization. Then, dissipation increases slightly back for the next 100 min and subsequently maintains a linear growth, with extremely low steepness, until Δ*D* = 3 × 10^−6^. The final rinse with buffer caused the drop of that value to a half.

**Figure 3:**
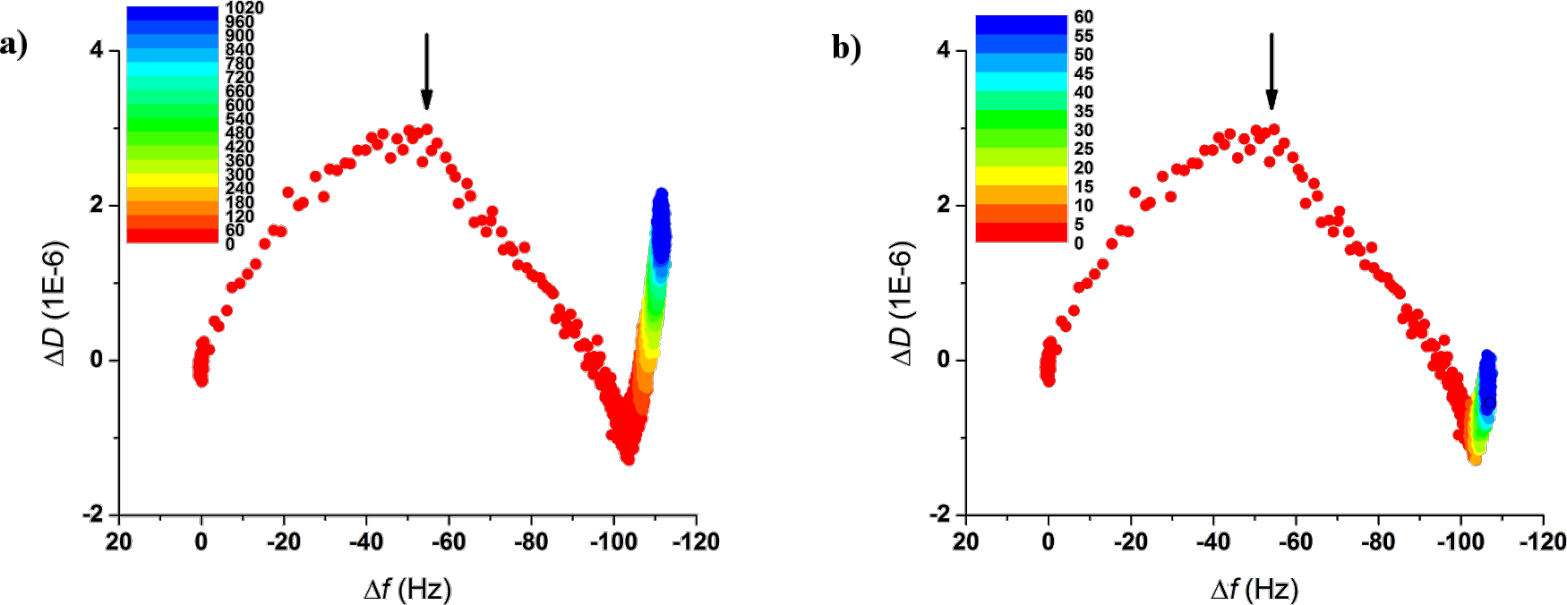
*Df* plots representing the binding of SbpA onto the SCWP film. The color scales indicate the elapsed time after protein injection for (a) the full incubation process (1020 min, color step 60 min) and (b) the initial 60 min of the process (5 min color steps). Black arrows indicate the transition point towards crystalline state.

A better way to follow the concomitant evolution of the protein layer in terms of bound mass and viscoelasticity of the film is by means of the so-called *Df* plots, as seen in Figure 3. It must be noted that the starting coordinates (0,0) were adapted to exactly refer to the moment at which SbpA is injected into the chamber while the red-to-blue color variation reflects the elapsed time, as denoted by the color scale on the inset. Thus, Figure 3a shows the increasing trend of Δ*D*, up to a maximum value (3.5 × 10^−6^) is only observed for the initial variation of 50–60 Hz. After this maximum value, the subsequent SbpA binding is followed by an almost linear drop in Δ*D* down to values even below the starting value. This is only possible because of the softness of the layer underneath, which might collapse slightly upon S-layer deposition on top. At a frequency variation of −100 Hz, the process seems to reach a second stage after which no additional protein is bound, but only Δ*D* undergoes a change. The vertically increasing dissipation might correspond to a slow system rearrangement caused by the simultaneous motility of the supporting SCWP film and the lateral reorganization of the S-layer, both leading to the final structure. While both the film formation and recrystallization-induced hardening take place in the initial 60 min of the incubation, it takes around 16 h for the gradual film adjustment. Indeed, as shown in Figure 3b, the S-layer formation can be assumed to be almost completed within the first 15 min. These results regarding SbpA recrystallization on a nature-mimicking SCWP film will be considered for comparative and descriptive purposes in the following section.

### S-layer formation on hydrophobic and hydrophilic SiO_2_

**Figure 4:**
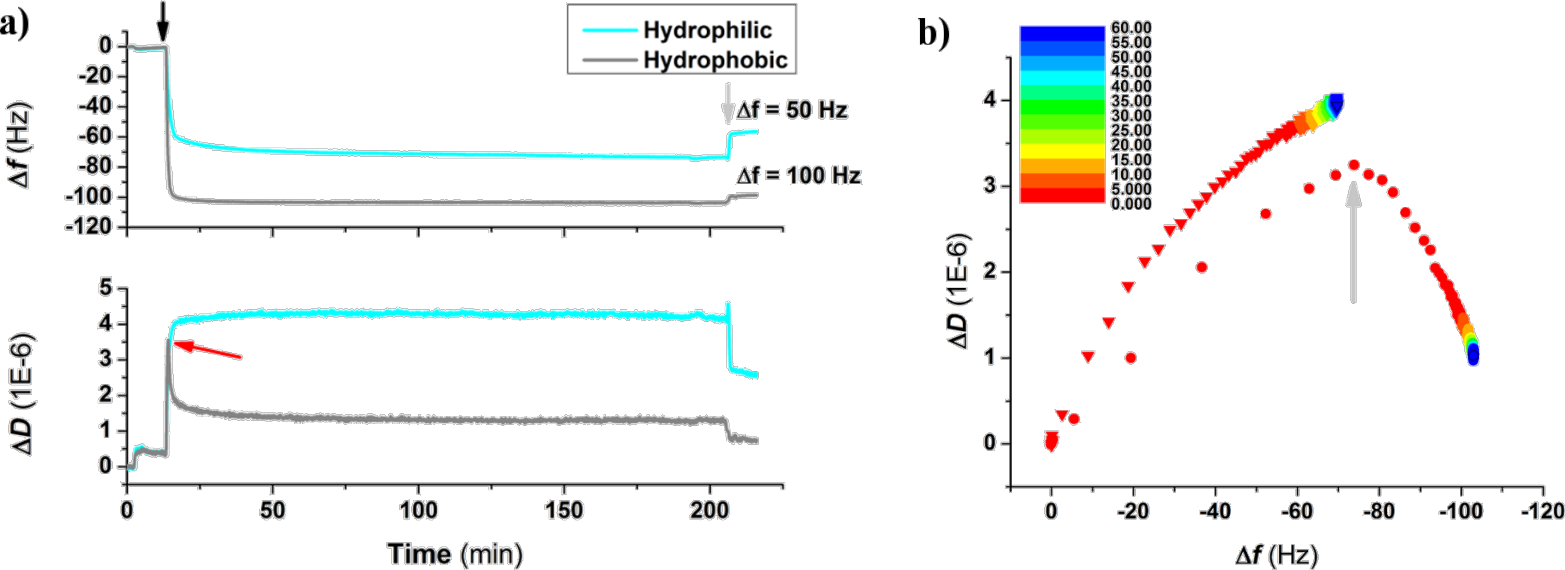
(a) Time evolution of Δ*f* and Δ*D* for the injection of SbpA protein on both hydrophilic and hydrophobic SiO_2_ substrates. The black arrow indicates the exact moment of protein injection while the grey arrows refer to the buffer rinse at the end of the process. The red arrow points out the appearance of the peak in Δ*D* in the hydrophobic case. In (b), the *Df* plot comparing the binding of SbpA onto both substrates is shown for the initial 60 min of the process, as indicated by the color scale. Grey arrow indicates the transition point*.*

In order to investigate the impact that surface wettability has on the binding and recrystallization of SbpA in the absence of specific protein–substrate interactions, the above mentioned protocol was again followed for silicon dioxide substrates under different wettability conditions: either UV/ozone-treated (hydrophilic, water contact angle Θ < 10°) or vapor-coated with a fluorosilane (Θ = 90°). Figure 4a depicts the time evolution of both Δ*f* and Δ*D* upon injection of 0.1 mg/mL SbpA in CB. It can be observed how from the very initial moments of the incubation, the behavior of SbpA is different on hydrophilic SiO_2_ substrates compared to that on hydrophobic fluorinated SiO_2_. A frequency plateau is reached in the former case after a decrease of only −60 Hz, while in the latter case it drops down to −100 Hz before stabilization takes place. In addition, recrystallization on the hydrophobic substrate is accompanied by the characteristic peak in dissipation but does not occur for the binding of SbpA to a hydrophilic sample. After 3 h incubation and attending to unvarying Δ*f* and Δ*D* values, especially in the fluorinated substrate, the process was stopped by flushing of CB. It is already well known by the authors that long incubation (>15 h) of SbpA on hydrophilic substrates leads to the formation of a crystal-like film. However, because of the lack of resemblance to the natural case (in bacteria), these results were not considered for further comparison. However, similar results to those of the hydrophobic substrate in terms of frequency variation, as well as the presence of the structural transition peak in Δ*D*, have already been reported in previous studies as developed by our group for different types of hydrophobic alkylsilanes (octadecyltrichlorosilane) [17] and alkylthiols (dodecanethiol and hexanethiol) [18] on silica and gold, respectively. In those cases the real-time analysis differed significantly from the one carried out here.

Figure 4b shows the corresponding *Df* plot for the initial 60 min of the two processes described above and indicates the surface wettability-dependent trend. SbpA binding on the hydrophobic surface repeats the 2-step formation seen for SCWP, with a clear transition peak taking place. In contrast, the binding to hydrophilic SiO_2_ shows a much slower process, with no characteristic transition peak in it, and what seems to be a still ongoing dissipation increase.

### Comparison between SCWP and hydrophobic SiO_2_

A quick glimpse at the QCM-D results discussed above shows the high similarity between the recrystallization processes on both the nature-mimicking SCWP and the hydrophobic SiO_2_. This is a remarkable outcome: The natural case is featured by, among others, specific protein–carbohydrate interactions involved in the S-layer formation [25], while on fluorinated-SiO_2_ the protein binding and crystal formation is mainly driven by hydrophobic interactions (non-specific). Also, SCWP shows a rather hydrophilic behavior (contact angle Θ < 10°), opposite to that of the fluorosilane. However, and in addition to the transition peak shown in Δ*D* and the final SbpA mass bound, the rapidity by which the S-layer formation process reaches completion on both surfaces is practically the same.

**Figure 5:**
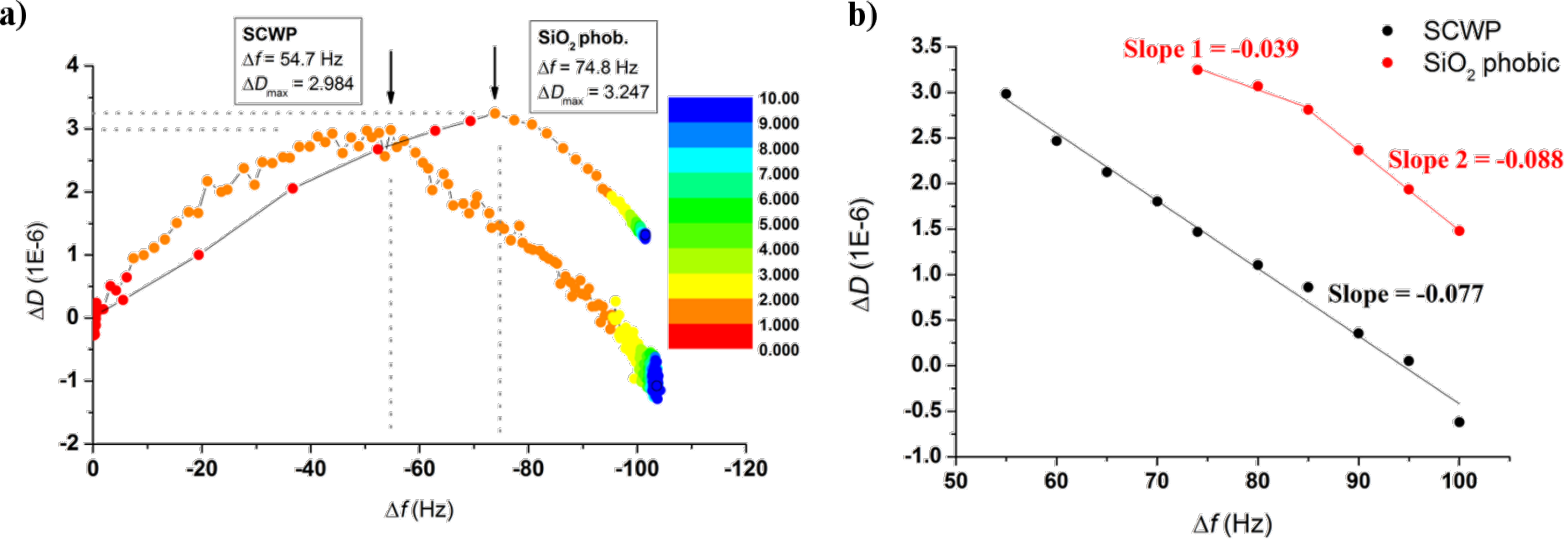
(a) *Df* plot comparing the binding of SbpA onto SCWP and hydrophobic SiO_2_ for the initial 10 min. (b) *Df* slope evolution after achievement of the maximum value of Δ*D*.

In Figure 5a the evolution of the recrystallization process for the initial 10 min is compared for both using the respective *Df* plots. As can be observed, besides the fact that both, the trend followed and the absolute values reached (including the same times required for formation and the Δ*D* maximum values at the transition peak), are certainly comparable. However, a deeper analysis highlights the existence of a few differences: Firstly, the position at which the transition towards a higher rigidness takes place is shifted in frequency. This means that in the case of the SCWP a lower amount of mass needs to be bound (55% of the total mass). For fluorinated substrates almost the 75% of the mass is required before the observation of the characteristic peak. Secondly, the capability of S-layers to rearrange after complete SbpA deposition changes from one system to the other. As mentioned above, the S-layer on top of the SCWP seems to have the ability of undergoing a rearrangement, which is reflected by a gradually increasing dissipation as the system is left to evolve. Such behavior already appears within the first 60 min of incubation, as indicated by the final slope in Figure 3b. On the contrary, recrystallization on hydrophobic SiO_2_ shows no trace of final rearrangement after the films is formed.

The most plausible explanation for these points relies on two factors, namely, the motility of the underlying layer and that of the protein film, which regulate the capability of the system to minimize the energy and lead to the mentioned differences in the final layout. For a fluorosilane coating underneath, the standing positions of the S-layer are somehow fixed and the lateral motion of the film is restricted. This is completely opposite in the case of a SCWP-coated substrate. This is probably better understood during long incubations, but also seems to play a key role in the very first moments. A good example of such influence can be seen in Figure 5b. The slope measured immediately after the transition peak in SCWP is twice of that of SiO_2_, and stays constant along the rest of the process, while the slope in the latter only drops for the last 15% of the S-layer formation. Although the SbpA adsorption process requires only about 2 min in both examples, the substrate motility leads to achievement of lower Δ*D* values on SCWP because of this concomitant re-organization as SbpA attaches, in addition to the specific SbpA–SCWP interactions. As a result, also the number of SbpA units required to start the transition towards crystal formation decreases, as the process (i.e., the protein–protein contact) is favored by the polymer below.

**Figure 6:**
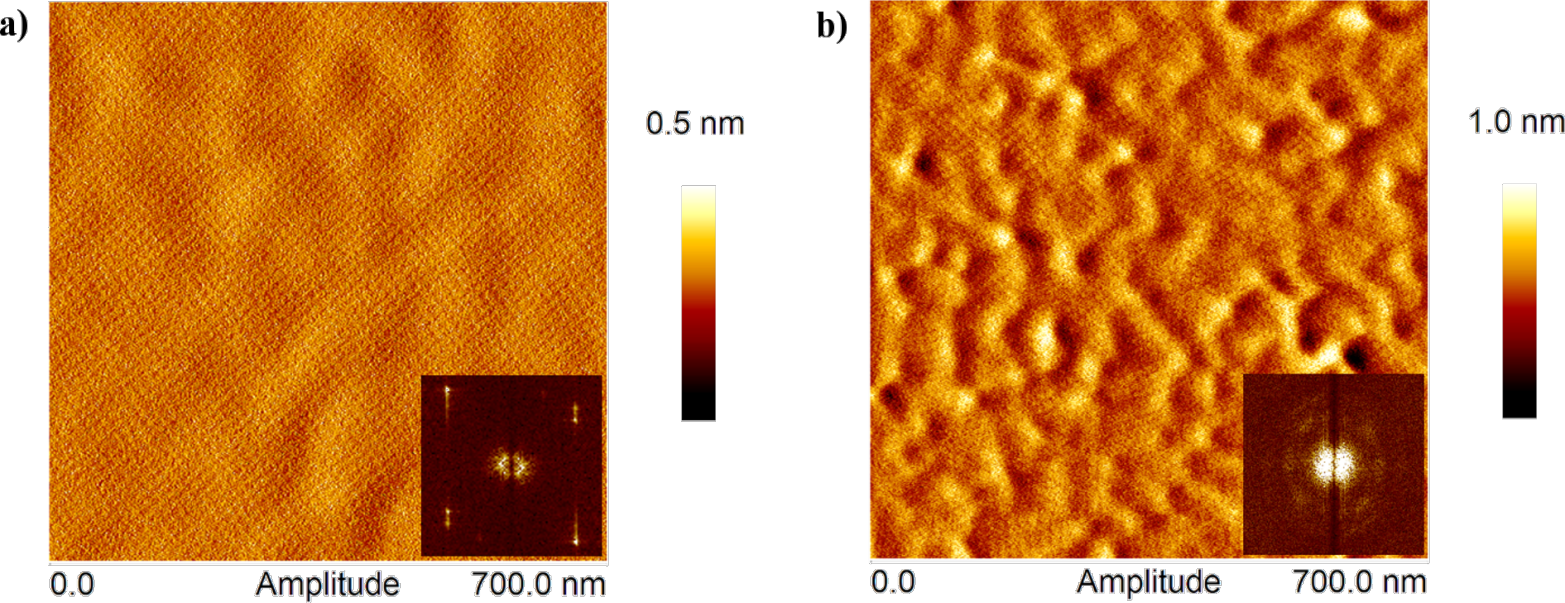
Atomic force amplitude micrographs (700 × 700 nm^2^) showing the surface topography of SbpA recrystallized on (a) secondary cell-wall polymer and (b) fluorosilane-modified hydrophobic SiO_2_. Bottom insets show the fast Fourier transform images of the crystalline structures formed.

In terms of the final topography, the atomic force micrographs shown in Figure 6 confirm what was deduced from the QCM-D results. The S-layer on top of the SCWP appears as an even coating consisting of a boundary-free crystalline domain (Figure 6a), in contrast to the multiple small domains forming the S-layer on fluorosilane with visible physical boundaries between them (Figure 6b). The included fast Fourier transform (FFT) images contribute to visualize the uniformity of the crystalline layers. For SCWP the FFT appears as a well-defined square, resulting from a unique crystal orientation while the halo observed for hydrophobic substrates derives from the multiple crystal orientations. In both cases the measured lattice spacing was 13 nm from center to center. Therefore, the continuous re-organization of the crystalline film as it is formed, allowed by the motility of the supporting polymer below, leads to a very homogeneous S-layer, of high resemblance to the one found on bacterial surfaces [1].

## Conclusion

S-layer recrystallization of SbpA bacterial protein has been shown to be highly influenced by surface wettability of the supporting substrate. Characterization of the recrystallization process on top of both hydrophilic and fluorosilane-modified hydrophobic SiO_2_ surfaces led to large differences in terms of process kinetics and development, as observed from the respective real-time physicochemical analysis. S-layer membrane formation on a hydrophobic sample resembled more faithfully the behavior of nature-mimicking secondary cell-wall polymer films, including the intermediate transition stages, besides the existing differences between both substrates. However, the fluid character of the underlying SCWP has a strong impact on the lateral motility as SbpA binds and enables a different final arrangement of the coating.

## Experimental

### Materials

Bacterial cell-surface layer protein, SbpA (*M*_w_ = 120 kDa) was isolated and purified from *L. Sphaericus CCM 2177* following the standard protocols [26]. Protein recrystallization buffer was prepared with 0.5 mM Trizma base (Sigma, Germany) and 10 mM CaCl_2_ (98% Sigma-Aldrich, Germany) and adjusted to pH 9.

### Sample preparation

**S-protein preparation:** After isolation, the protein solution was centrifuged at 5000 rpm for 5 min in order to separate the S-protein monomers from self-assembly products and was stored at 4 °C. Then, before each experiment, the supernatant solution was diluted using the appropriate amount of recrystallization buffer to a final concentration of 0.1 mg/mL.

**Assembly of fluorinated silane:** Experiments were performed either ex situ onto silicon wafers or in situ onto QSX303 SiO_2_-coated quartz sensors (4.95 MHz, Q-sense AB, Sweden). Both substrates were pre-treated with UV/ozone (BioForce Nanosciences, Ames, IA, USA) for 30 min and then placed overnight in an evacuated desiccator with ca. 30 µL of 1*H*,1*H*,2*H*,2*H*-perfluorododecyltrichlorosilane (Sigma Aldrich) in chloroform for vapor silanization. Finally, they were rinsed with ethanol and water, dried under N_2_ and immediately used in the next step.

**Secondary cell-wall polymer (SCWP) preparation:** SCWP was isolated from peptidoglycan-containing sacculi of *L. sphaericus CCM 2177* and purified [24]. Chemical modification of the reducing end on the polymer chains and introduction of a terminal sulfhydryl group by modification with 2-iminothiolane was performed [3]. Subsequently, thiol–secondary cell-wall polymer (thiol-SWCP) was used to activate gold surfaces, either QSX301 gold sensors (4.95 MHz, QSense AB, Sweden) by means of peristaltic pumping (SM935C, Ismatec, Zürich, Switzerland), in QCM-D, or commercial flat gold Arrandee^TM^ chips (Arrandee Metal GmbH, Germany) by immersion into a petri-dish filled with thiol-SCWP solution for AFM measuring purposes. After one hour of incubation we obtained a SCWP surface for S-protein recrystallization.

### Methods

**Atomic force microscopy (AFM):** Recrystallized proteins were visualized using a multimode-AFM (Bruker AXS, Santa Barbara, USA) controlled by a Nanoscope V equipped with a J-scanner. Silicon-nitride probes (DNP-S, Bruker, USA) with a spring constant of about 0.3 N/m were used in the experiments, which were calibrated on SiO_2_ using the thermal method. Prior to its use in the AFM fluid cell, the cantilever was cleaned with UV/ozone for 20 min. Once mounted, the system was kept immersed in ultrapure water until stabilization of the deflection signal. Data acquisition was carried out in tapping mode, in order to not disturb the structure and at a scan rate lower than 2 Hz.

The solutions containing recrystallization buffer and SbpA were injected into a chamber sealed by a silicone O-ring with a syringe. Before use, the fluid cell, tubing, and O-rings were washed overnight with 2% SDS, rinsed gently with ultrapure water, and dried with nitrogen. All images were processed with the Nanoscope program.

**Quartz crystal microbalance with dissipation (QCM-D):** QCM-D experiments were performed in a Q-Sense E4 instrument (Q-Sense AB, Sweden). Prior to their use, gold-coated quartz sensors (for thiol–SCWP binding) were sonicated in 2% (w/w) SDS solution for 20 min and then rinsed with ultrapure water and ethanol. The crystals were dried under N_2_ stream, treated with UV/ozone for 30 min and mounted into the QCM-D chamber. For measurements involving the use of hydrophobic SiO_2_ sensors, these were treated as explained above. The same protocol was applied to hydrophilic SiO_2_ sensors with the exception of the overnight vapor deposition.

All experiments were performed at 25 °C. Real-time variations of Frequency (Δ*f*) and dissipation (Δ*D*) parameters were observed at several overtones (*n* = 3, 5, 7,...,13) throughout the QCM-D experiment. Additionally, the Δ*D*-versus-Δ*f* plot can also be used to analyze QCM-D data. Each point of a Δ*D*–Δ*f* plot represents a dissipation and frequency date at a certain time that provides a more detailed view on the viscoelastic evolution of films per mass unit (Δ*m*) change. The shape of the Δ*D*–Δ*f* plot, as well as the slopes derived, can characterize specifically one type of process and enables the differentiation between them.

**Contact angle measurements:** Sessile-drop experiments were performed with a contact-angle measuring system (Kruss D100, Hamburg, Germany). Millipore water (18.2 MΩ·cm) was used as the liquid phase. Water drops (ca. 10 µL) were deposited on the substrates and the contact angles were obtained from the drop profiles.
